# Narrative comprehension beyond language: Common brain networks activated by a movie and its script

**DOI:** 10.1371/journal.pone.0200134

**Published:** 2018-07-03

**Authors:** Pia Tikka, Janne Kauttonen, Yevhen Hlushchuk

**Affiliations:** 1 Department of Media, Aalto University School of Arts, Design and Architecture, Helsinki, Finland; 2 Baltic Film, Media, Arts and Communication School, Tallinn University, Tallinn, Estonia; 3 Department of Neuroscience and Biomedical Engineering, Aalto University School of Science, Espoo, Finland; 4 NeuroLab, Laurea University of Applied Sciences, Espoo, Finland; 5 Advanced Magnetic Imaging Centre, Aalto NeuroImaging, Aalto University School of Science, Espoo, Finland; 6 HUS Medical Imaging Center, Radiology, University of Helsinki and Helsinki University Hospital, Helsinki, Finland; University of Cambridge, UNITED KINGDOM

## Abstract

Narratives surround us in our everyday life in different forms. In the sensory brain areas, the processing of narratives is dependent on the media of presentation, be that in audiovisual or written form. However, little is known of the brain areas that process complex narrative content mediated by various forms. To isolate these regions, we looked for the functional networks reacting in a similar manner to the same narrative content despite different media of presentation. We collected 3-T fMRI whole brain data from 31 healthy human adults during two separate runs when they were either viewing a movie or reading its screenplay text. The independent component analysis (ICA) was used to separate 40 components. By correlating the components’ time-courses between the two different media conditions, we could isolate 5 functional networks that particularly related to the same narrative content. These TOP-5 components with the highest correlation covered fronto-temporal, parietal, and occipital areas with no major involvement of primary visual or auditory cortices. Interestingly, the top-ranked network with highest modality-invariance also correlated negatively with the dialogue predictor, thus pinpointing that narrative comprehension entails processes that are not language-reliant. In summary, our novel experiment design provided new insight into narrative comprehension networks across modalities.

## Introduction

*A young girl Nora stares shocked at her mother Anu*. *Anu stands expressionless by the kitchen table and scrapes the left-over spaghetti from Nora's plate into a plastic bag*. *She places the plate into the bag and starts putting there other dining dishes*, *takes a firm hold of the bag and smashes it against the table*. *Nora is horrified*: *"Mother*! *What are you doing*?*”*. *Anu continues smashing the bag without paying attention to her daughter*. *Nora begs her to stop*. *Anu collapses crying against the table top*. *Nora approaches*, *puts her arms around the crying mother and starts slowly caressing her hair*.

The dramatic scene describes a daughter witnessing a nervous breakdown of her mother. Its narrative content remains the same should one read it in a textual form or viewed it as a movie. It is relatively well known how narratives are processed in the distinct human sensory cortices depending on the sensory input through which the narrative is perceived (reading, listening, viewing; [1–5]). However, far less is known of how the human brain processes meaningful narrative content *independent* of the media of presentation. To tackle this classical dichotomy issue between form and content in neuroimaging terms, we employed functional magnetic resonance imaging (fMRI) to provide new insights into brain networks relating to a particular narrative content while overlooking its form.

To our best knowledge, none of the previous fMRI studies have focused on the question of how similarly responds the human brain to the same dramatically composed events perceived freely in textual versus audiovisual form. So far, only a few fMRI studies have compared how the subjects respond to the *same* story content in two different linguistic conditions, when reading and listening to the same narrative [6], or listening to the same narrative in two different languages [7]. Going beyond these previous language-based studies, we presented the same drama content in two forms that differed to a greater extent since only one of them relied exclusively on verbal communication (written language): All subjects both viewed a short film and read its screenplay during fMRI measurement. Our hypothesis was that narrative-related brain activity would temporally correlate across the two conditions due to synchrony of presented narrative events despite the distinct forms of presentation. Major narrative events occurring at specific timepoints, such as new information, character interactions and plot twists, are not bound to specific media of presentation. Neural responses to such events are not expected to be instant, but instead results from accumulated information and inference about the plot (see, e.g., [8]). One may therefore expect that even if the media is different, a compelling and coherent narrative will regardless lead to synchronized neural activity on longer timescales, e.g., few minutes.

Our method of choice was independent component analysis (ICA) that is a multivariate data-driven dimension reduction method for distinguishing a set of independent functional brain networks [9]. ICA is particularly useful for continuous naturalistic stimuli that lacks tightly controlled structure, such as stimulus on/off blocks [10,11]. When compared to inter-subject correlation (ISC)—another popular data-driven analysis method operating on individual voxels—results of ICA are typically easier to interpret thanks to significantly smaller data dimensionality [11]. It’s also useful in whole brain exploratory analysis when no pre-defined regions of interest are used or available.

Previous studies have shown that processing of cinematic and audio narratives occurs in hierarchical manner so that coherent narrative segments are associated with increased inter-subject fMRI signal synchronization in ‘higher-order’ (e.g., frontal, temporal and superior parietal) regions compared to unstructured (e.g., scrambled) stimuli that only synchronizes lower-order sensory regions [3,5]. As the duration of the coherent stimulus increases, so does the spatial extend of synchronization in higher-order regions, thus implying the existence of hierarchical models of narrative comprehension. Furthermore, it has been demonstrated that certain key properties of movie narratives, such as plot suspense and cognitive demand, are highly correlated with activity in fronto-parietal networks [12]. In accordance with these previous results, we expected the modality-invariance to increase from the lower-order sensory regions towards high order cognitive regions in the parietal, temporal and frontal areas in the current study.

## Materials and methods

### Participants

We recruited 37 healthy right-handed Finnish-speaking adults after their informed consent. Due to excessive head movement, vigilance changes and certain technical issues the MRI data of 31 subjects were taken into the final analysis (13 females; mean age 27 years, range 19–53). Large sample size was considered important in minimizing inter-subject variations in personal reading and film-viewing practices, which were not directly controlled in the study. All subjects reported they had not seen the stimulus movie ‘Heartbeats’ before. The study received a prior approval from the Ethics Committee of Helsinki and Uusimaa Hospital District.

### Stimuli

#### Stimuli design

The experiment consisted of two functional runs: (1) "script" run (the screenplay text of the episode “Nora’s room”, divided into short one- or two-sentence text slides) and, (2) "movie" run containing the final filmed episode “Nora’s room” (see next subchapter for details on the cinematic material). Both movie and text slides were presented in Finnish and in counter-balanced manner with respect to stimulus order, i.e., movie was the first condition for half of the group (15 subjects).

In the "script" run we showed the subjects a sequence of short text slides, which eventually amounted up to a complete story, the same as in the filmic scene. The black-colored text appeared in the center of the slides with gray background. The length of the text in each slide was kept short to ensure readability while the duration of the corresponding events in the film scene (1-4s; average 3.13s) defined the slide duration. Such arrangement resulted in the synchronization of the text slides to the events in the film (relative to the beginning of the story in the corresponding run). For example, each dialogue in the screenplay was shown exactly at the same time from the beginning of the run as it would be heard/shown during viewing of the film. In similar manner, the action sentences were synchronized to the actions in the film. Consider, for example, “Nora looks at her mother” both as a written action as well as a filmic event. In this manner we could create identical synchronized tracks of stimulus of (1) written text and (2) film medium. As a result of this accurate synchronization of narrative events, we expected substantial synchronization to occur also for the neural activity in certain brain regions.

#### Cinematic material

We selected one episode from a Finnish drama film “Heartbeats” (“Kohtaamisia”, directed by Saara Cantell 2010). The episode involves three characters: a girl Nora (14 y; noted as N in the dialogues), mother Anu (42 y; A) and father Petri (42 y; P); it depicts a continuous 7 minutes' shot in an apartment. The film is shot with cinematographic single-take method, i.e., there are no cuts, or junctures, between shots, and thus it may engage the viewer’s attention in a fashion similar to natural perception as opposed to film episodes composed of edited cuts. With the single-take method the handheld camera fluently follows the events, for example, changing the framing of the three protagonists in a wide shot into an intimate facial close-up of one of them. The episode’s casual every-day life gradually develops into a psychological drama, leading to the emotionally loaded climax–the young girl witnessing the nervous breakdown of her mother. As the story progresses, it becomes evident–although never explicitly stated–that Petri is having an extramarital affair, which is a major factor for the dramatic ending.

#### Stimulus presentation

The subjects watched visual stimuli during the scanning sessions (free-viewing). The images were generated with a 3-digital light processor (DLP) data projector VistaPro, Electrohome Ltd. and projected to semitransparent screen attached behind the headcoil. The subjects observed the screen via a mirror at a viewing distance of 35 cm. The actual size of the observed film stimuli on the screen was approximately 23 cm (width) × 13 cm (height). The text stimuli were formatted to cover approximately the same width (the size of the font was however kept the same size for all the text slides). The gray screen with a fixation cross in the middle was shown in the beginning of each run until the end of the dummy scans’ acquisition. The presentation and timing of the stimuli were controlled by a personal computer running Windows Millenium and Presentation® software (Version 14.9, Neurobehavioral Systems Inc., Albany, CA).

### MRI data acquisition and analysis

#### MRI data acquisition

We acquired functional MRI (fMRI) data on a Signa HDxt 3T MR scanner (GE Healthcare Ltd.) using a gradient-echo planar imaging sequence with the following parameters: flip angle = 75°, repetition time (aka time-of-repeat, TR) = 2015 ms, echo time = 32 ms, field of view = 220 mm, matrix 64 × 64, altogether 40 axial-oblique slices (thickness 3.5 mm), and interleaved slice acquisition. Subsequent analysis excluded the first four (dummy) volumes from each run in order to avoid partial magnetic saturation effects.

Anatomical brain images were obtained in the sagittal plane with a 3-D fast spoiled gradient echo sequence (inversion-recovery prepared): flip angle = 15°, repetition time = 10 ms, echo time = 3 ms, field of view = 256 mm, matrix 256 × 256, slice thickness 1.0 mm. The acquisition of both anatomical and functional MRI images deployed ASSET parallel imaging option with the acceleration factor of 2.0.

We also employed MRI-compatible eye-tracking system (IVIEW X™ MRI-LR; SensoMotoric Instruments GmbH, Germany) to monitor subjects’ eye-movements and to ensure their vigilance throughout the fMRI runs.

#### MRI data preprocessing

Due to excessive head movement, vigilance changes and certain technical issues the MRI data of only 31 subjects were taken into the final analysis.

All data preprocessing was performed using in-house built pipeline for fMRI data analysis: fMRI Data Processing Assistant (fDPA; written by Eerik Puska and Yevhen Hlushchuk). It is a MATLAB (The MathWorks Inc., Natick, Massachusetts) toolbox based on SMP8 software (http://www.fil.ion.ucl.ac.uk/spm/software/spm8/) and Data Processing Assistant for Resting-State fMRI (DPARSF, V2.0_110505, http://www.restfmri.net; [13]). For dealing with artifacts fDPA encorporates functions of ArtRepair toolbox (http://cibsr.stanford.edu/tools/human-brain-project/artrepair-software.html; [14]) and DRIFTER toolbox (http://becs.aalto.fi/en/research/bayes/drifter; not used in this study).

First the fMRI data were realigned, coregistered to the anatomical scans and normalized to MNI space [15] using unified segmentation of T1-structurals (normalized voxel size 2 × 2 × 2mm^3^). Normalized fMRI data subsequently underwent volume artefact removal (thresholds used with ArtRepair: % threshold at 1.3, z-threshold at 2.5, movement threshold per volume at 0.5mm), spatial Gaussian smoothing at FWHM of 7mm and high-pass filtering at 0.01Hz. Quality of the preprocessed data was validated by computing and inspecting framewise displacement and DVARS time-courses [16].

#### Independent component analysis (ICA)

We further analyzed our data with spatial independent component analysis. For that we exploited GroupICATv2.0e (GIFTv1.3i) toolbox (http://icatb.sourceforge.net). Into ICA analysis we submitted 2 separate runs per subject: 212 volumes of fMRI data from the script-reading run and the same amount of the movie-viewing run, which ensured that components for both modalities were matched between both conditions and all subjects. The ICA estimated 40 independent components (ICs) using InfoMax algorithm with default settings and scaling of the components to percent signal change. For back-reconstruction of individual components at subject-level we utilized GICA3 which is preferred over GICA1 and GICA2 (detailed reasons for this choice see in Appendix A of [10]). The spatial maps of the back-reconstructed subject-level components were averaged across runs, which produced 31 subject-level spatial maps per component (i.e., 40 components per subject). Prior to averaging, we verified spatial similarity of back-reconstructed maps between conditions by computing full pair-wise, between-condition spatial correlation tensor over all maps (i.e., 31×40×40 = 49600 values). Out of these values, 1240 correspond to a situation where components are correctly matched between conditions (i.e., 31×40), while other correspond to incorrectly matched component pairs. As the spatial ICA maximizes spatial independence of components, latter values are assumed to be notably lower than the former [9].

After averaging across conditions (as implemented in GIFT toolbox), subject-level maps were assumed independent and transferred into SPM8 for the 2nd-level statistics (one-sample t-test with 30 degrees of freedom). The resulting maps were thresholded at p<0.001 (height threshold) with family-wise-error-rate (FWE) correction for multiple testing and spatial extent threshold of clusters (i.e., spatially connected voxels) set to 50 normalized voxels. For anatomical labeling of the areas within component clusters we exploited AAL atlas [17].

#### Correlation of ICs with a dialog regressor

ICA time-courses were compared against dialog time-courses that correspond to presence of dialog in the stimuli (spoken or written). As the dialog is a key element in the story, conveying both narrative and emotional tension, it is likely to be temporally correlated with one or several IC time-courses. First a Boolean (on/off) type dialog envelope was extracted from the stimuli and convolved with a standard haemodynamic response function (HRF; function *spm_hrf* in SPM), then the regressor was interpolated and high-pass filtered (0.01Hz cut-off) to match timing and frequency of the ICs. All IC time-courses in both conditions (i.e., total 40+40 = 80) were correlated against the dialog regressor and mean correlation values were compared against permutation distributions by taking a percentile. This resulted in approximated two-tailed p-values for all ICs in both conditions. Empirical null-distributions were collected by computing all correlation values for the shift-permuted dialog regressor. Iterating through all 209 shifts (with minimum shift of 2 TRs) and all 40 ICs, resulted in total 8360 (i.e., 209×40) correlations in the null-distribution. The same permutation scheme was also used in cross-modal comparisons for IC time-courses to estimate statistical significance of temporal correlations. To ensure meaningful interpretation of the correlation sign, all IC time-courses were compared against the original preprocessed fMRI data (time-course averaged over 500 voxels around the peak of a component) to validate their signs and no need for sign adjustment was detected.

## Results

### Quality control of the data

No excessive spiking was present in the framewise displacement and DVARS time-courses for the 31 datasets used in the data-analysis. Root-mean-squared (RMS) values were 0.119 mm (mean over subjects) for the framewise displacement and 0.482 (mean over subjects) for DVARS. One subject had a single framewise displacement peak over 2 mm, but the data was deemed suitable for the analysis after using ArtRepair correction. No significant difference in head motion was present between movie and script conditions (p = 0.85 for framewise displacement RMS; paired-sample t-test).

### Isolating narrative networks (ICs)

The spatial ICA estimated 40 independent components common for the "script" and "movie" runs. Between these two conditions mean spatial correlation of back-reconstructed maps over subjects was 0.65 (SD 0.07; 40 values) for the matched components (i.e., the same component in both conditions), while the mean correlation was 0.00 (SD 0.04; 1560 values) for other component pairs. This ensured that maps between conditions were similar enough for proceeding with the subject-wise averaging of the maps across conditions.

Our study focused on identifying narrative-related brain networks that are in play when people are reading narrative text or viewing the same narrative as a movie. To follow the unfolding of the story (textual or audiovisual), the cognitive processes of narrative comprehension require keeping in mind the past events as well as anticipating the future events.

Due to the synchronization of the audiovisual and textual narratives so that the character actions and dialogues followed the same timeline during these otherwise very dissimilar stimuli, we expected narrative-related brain activations to feature similar time-course. Hence, to reveal, which ones of the independent component networks were most similar in the two conditions, we calculated the correlation coefficient between their group-averaged time-courses (“correlation over averages”) and ranked the ICs accordingly (*ranking A*). These correlations varied between -0.12 and 0.71 (mean 0.28 with SD 0.17) with the highest 22 being statistically significant against empirical null-distribution (two-tailed p<0.05, FDR adjusted over 40 components). An alternative ranking, based on the average of subject-level inter-condition correlations (“average over correlations”), resulted in different ranking (*ranking B*). These correlations were lower and varied between -0.01–0.19 (mean 0.07 with SD 0.05) with the highest 16 being statistically significant against empirical null-distribution (two-tailed p<0.05, FDR adjusted over 40 components). When expressed in ranking A, the first 10 components from ranking B were (from highest to lowest): 4, 17, 1, 8, 3, 2, 5, 11, 25 and 24, i.e., the component with the highest correlation in ranking A was the 3^rd^ highest in ranking B etc. Total 5 out of 7 top components were shared between the two ranking systems and these 5 components also surpassed all values in empirical null-distribution for ranking A. At p<0.05, total 13 components were statistically significant in *both* ranking systems. As we were only interested in activation similarities between modalities (not individual subjects), we deemed ranking A more suitable for the current study. With modality-wise averaging and the relatively large group size of 31 subjects, ranking A should effectively minimize contamination by the subject-specific intrinsic signals.

**Fig 1:**
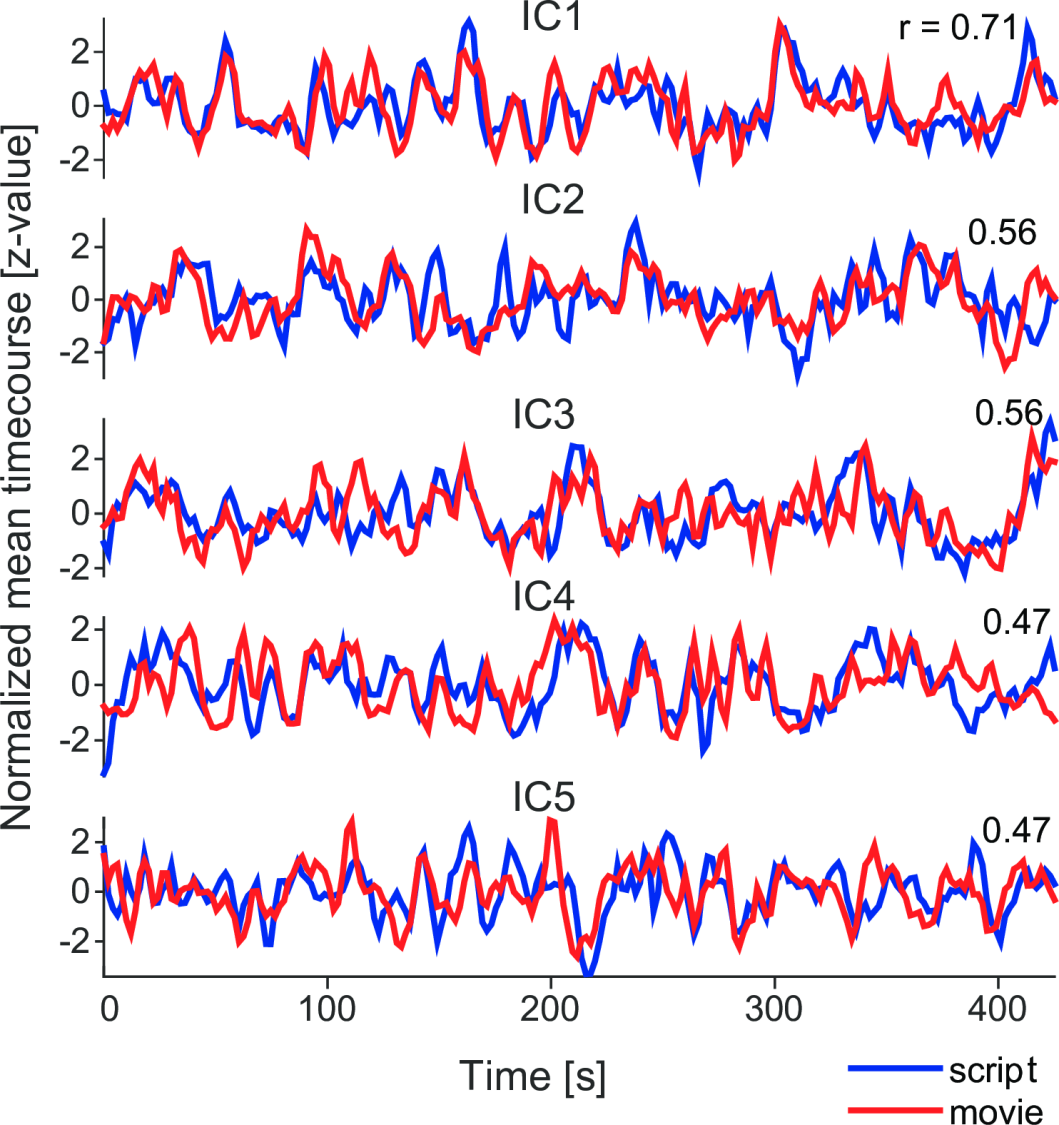
Time-courses of TOP-5. Normalized mean time-courses of TOP-5 ICs arranged from IC1 (top row; correlation 0.71) to IC5 (bottom row; correlation 0.47) featuring the highest cross-correlations of the time-course between movie (red) and script (blue) narrative presentation forms.

**Fig 2:**
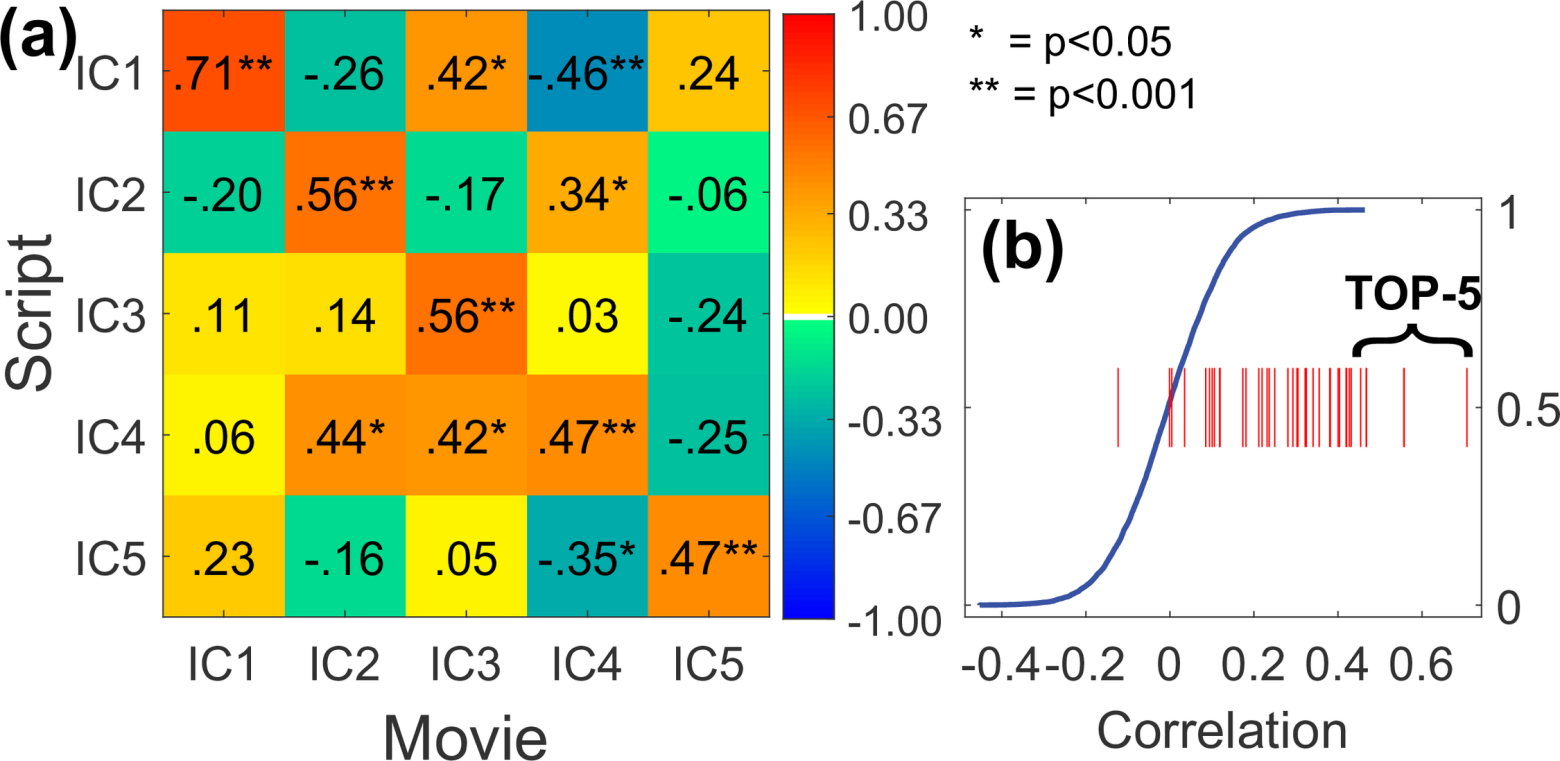
Temporal correlations between movie and script conditions for TOP-5 components. (a): Each row (column) corresponds to a group-averaged IC time-courses for the script (movie) condition. Statistically significant correlation coefficients are marked with stars (p<0.05 and p<0.001; FDR adjusted over 5×5 = 25 elements). (b) Component-wise matched correlation coefficients (red lines; one for each component, 40 values) plotted against the cumulative empirical null-distribution (blue line). Highest five correlations correspond to TOP-5.

**Fig 3:**
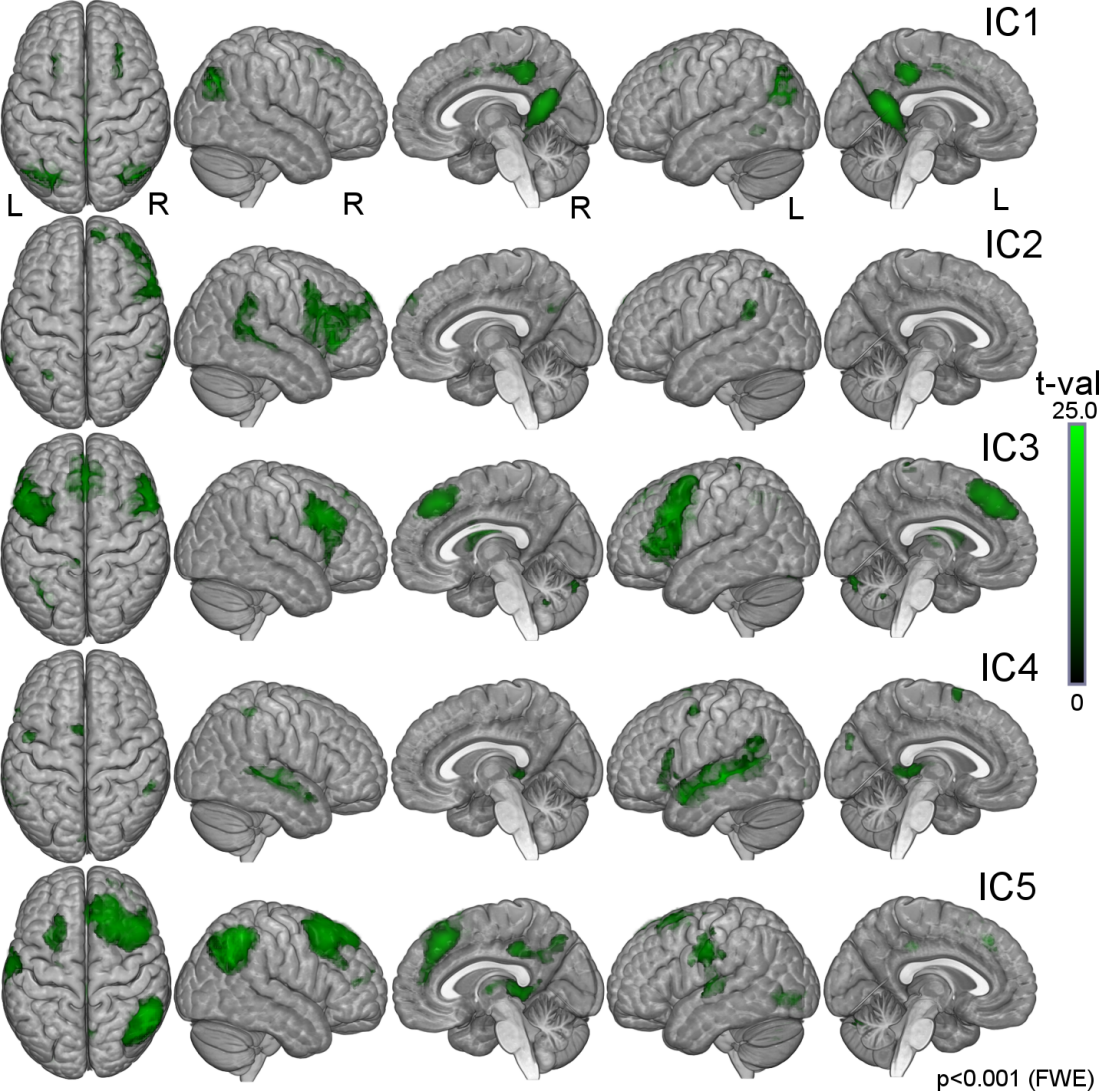
Visualization of TOP-5. Spatial t-value maps of TOP-5 ICs sorted from IC1 (top row) to IC5 (bottom row) and overlaid on partially transparent 3D brain template. Statistical threshold is set to p<0.001 (FWE) with the minimum cluster extent of 50 normalized voxels.

We chose top five components from ranking A for further investigation. Fig 1 depicts the time-courses for these five ICs with the highest correlation between the group-average time-courses of film-viewing and script-reading runs. These ICs were deemed narrative-related. The TOP-5 *narrative* components were labeled as IC1 (0.71; correlation coefficient), IC2 (0.56), IC3 (0.56), IC4 (0.47) and IC5 (0.47). All pair-wise temporal correlations between TOP-5 components (i.e., 5×5 = 25 values), all 40 between-condition correlation coefficients and the empirical null-distribution are depicted in Fig 2. Spatial locations of these five components are listed in Table 1 and depicted in Fig 3.

**Table 1:** Clusters of TOP-5. Anatomical labeling of the clusters of TOP-5 ICs at the statistical threshold p<0.001 (FWE). Only the major anatomical labels contributing at least 100 normalized voxels to a cluster are shown in the order of their size. The table lists such anatomical labels for each cluster until cumulative 75% of all voxels in the corresponding cluster is reached.

	Voxels	Peak			AAL label		Voxels	Peak			AAL label
		x	y	z				x	y	z	
**IC1**	6619	-42	-72	30	Occipital_Mid_L (16%)	**IC3**	3074	50	8	30	Frontal_Inf_Tri_R (27%)
					Precuneus_R (10%)						Frontal_Mid_R (26%)
					Precuneus_L (8%)						Frontal_Inf_Oper_R (21%)
					Calcarine_L (7%)						Precentral_R (17%)
					Angular_L (6%)		575	-30	-68	42	Parietal_Inf_L (66%)
					Calcarine_R (5%)		162	12	-80	-28	Cerebelum_Crus1_R (78%)
					Occipital_Sup_L (4%)		240	-16	-82	-24	Cerebelum_Crus1_L (69%)
					ParaHippocampal_L (3%)		454	-16	4	14	Caudate_L (49%)
					ParaHippocampal_R (3%)						Thalamus_L (24%)
					Cuneus_L (3%)		441	20	-6	22	Caudate_R (55%)
					Parietal_Sup_L (3%)	**IC4**	4665	-56	-24	0	Temporal_Mid_L (48%)
					Fusiform_L (3%)						Temporal_Sup_L (26%)
					Parietal_Inf_L (3%)						Frontal_Inf_Tri_L (5%)
	1199	40	-76	34	Occipital_Mid_R (46%)		1589	56	-6	-2	Temporal_Sup_R (64%)
					Angular_R (33%)						Temporal_Mid_R (27%)
	1386	-6	-34	42	Cingulum_Mid_L (40%)		440	-12	-32	4	Thalamus_L (43%)
					Cingulum_Mid_R (35%)		115	-4	6	68	Supp_Motor_Area_L (92%)
	230	28	32	52	Frontal_Sup_R (63%)	**IC5**	3152	50	-50	50	Angular_R (45%)
	218	-22	16	52	Frontal_Mid_L (79%)						Parietal_Inf_R (28%)
**IC2**	4408	52	32	-2	Frontal_Inf_Tri_R (27%)						SupraMarginal_R (8%)
					Frontal_Mid_R (20%)		5500	12	30	50	Frontal_Mid_R (30%)
					Frontal_Inf_Oper_R (19%)						Frontal_Sup_R (25%)
					Precentral_R (11%)						Frontal_Sup_Medial_R (14%)
	1981	56	-42	8	Temporal_Mid_R (40%)						Supp_Motor_Area_R (5%)
					Temporal_Sup_R (26%)						Cingulum_Ant_R (4%)
					SupraMarginal_R (22%)		1113	-62	-10	36	Temporal_Sup_L (35%)
	216	10	-64	32	Precuneus_R (78%)						Postcentral_L (33%)
**IC3**	7986	-46	20	30	Frontal_Inf_Tri_L (21%)		580	-14	-78	-28	Cerebelum_Crus1_L (78%)
					Frontal_Mid_L (17%)		190	30	56	12	Frontal_Mid_R (60%)
					Precentral_L (14%)		606	2	-30	38	Cingulum_Mid_R (47%)
					Frontal_Sup_Medial_L (13%)						Precuneus_R (38%)
					Frontal_Inf_Oper_L (9%)		585	10	-30	4	Thalamus_R (37%)
					Frontal_Sup_Medial_R (7%)		651	-32	14	58	Frontal_Mid_L (54%)
											Frontal_Sup_L (34%)
							734	-26	-88	-2	Occipital_Mid_L (51%)
											Occipital_Inf_L (40%)

Finally, as a comparison for TOP-5, we computed corresponding correlation coefficients for primary visual and auditory cortices for group-averaged BOLD signals (i.e., ranking A method). For four visual cortex ICs covering occipital lobe (identified by GIFT toolbox’s *network labeler* tool), correlations were notably lower at 0.32 (rank 17 out of 40), 0.25 (rank 23), 0.23 (rank 25) and 0.16 (rank 29). Similarly, for eight AAL atlas regions-of-interest (ROIs) covering occipital lobe, correlations between group-averaged mean BOLD signals were between 0.13 (rank 46 out of 116) and 0.35 (rank 108). For the primary auditory cortices results were similar with correlations 0.09 (ICA; rank 36 out of 40), 0.25 (AAL atlas, right hemisphere; rank 87 out of 116) and 0.26 (AAL atlas, left hemisphere; rank 80). Means of all correlation coefficients (i.e., ICs and AAL atlas ROIs) for group-averaged signals were positive (0.32 and 0.28). This was reflected by the fact that also the group-averaged global BOLD signal correlation over all voxels in the group mask (172419 normalized voxels) was 0.37.

#### IC1

IC1 component appeared bilateral and relatively symmetrical with a slight left dominance (Fig 2). Its biggest cluster covered the posterior parietal areas up to angular gyrus laterally and was contiguous with the bilateral activation cluster covering all but the superior part of the parieto-occipital sulcus (POS) and extending to hippocampus bilaterally. A corresponding posterior parietal cluster on the right appeared separate from the bilateral POS-hippocampus cluster at the current threshold.

The third cluster lies bilaterally at the border of posterior cingulate and anterior precuneus with slight extension into the middle cingulate. The above mentioned clusters correspond to certain components of hippocampocortical/default network [18]. The fourth and fifth clusters are situated in the superior frontal sulcus somewhat anterior to the precentral sulcus. Also this component contained a cluster in the right temporal cluster corresponding by the location to the medial superior temporal area (MST; [19]).

#### IC2

IC2 component covered mainly areas of the right hemisphere with the biggest cluster covering the inferior and partly middle frontal gyrus and extending to the anterior insula. The component also encompassed cluster in the right STS, region considered to be involved in biological motion/action recognition extending to the multisensory region of the supramarginal gyrus/posterior temporal operculum implied in action recognition [20]. Smaller clusters on the right lay in the dorsal precuneus and medial superior prefrontal cortex. This component encompassed only 2 minor clusters on the left: one in the supramarginal gyrus/posterior temporal operculum and another in the superior parietal lobule.

#### IC3

IC3 component is bilateral and relatively symmetric. The biggest 2 clusters of this component covered premotor areas (i.e. posterior parts of the middle and inferior frontal gyri). The right cluster was bigger and appeared contiguous with the bilateral activation in the dorsomedial prefrontal cortex (DMPFC). Smaller clusters were located in the left inferior parietal lobule, left and right cerebellum, caudate bilaterally and the left thalamus.

#### IC4

This component was relatively symmetrical, slightly left lateralized. Its biggest cluster spread along the left superior temporal sulcus (STS) extending to the pars triancularis of the left inferior frontal gyrus (IFG). The second size cluster spread along the right STS. Smaller clusters were located in the left thalamus, left supplementary motor area (SMA). Clusters in the left precentral cortex, right IPL, left cuneus and left inferior occipital cortex failed to exceed 100 normalized voxels in size.

#### IC5

This component is right lateralized. The cluster containing the component’s global peak covered mainly the angular gyrus in the right IPL. The component’s largest cluster covered the right dorsolateral prefrontal extending to the medial surface of the superior frontal gyrus. The third by size cluster extended from the left superior temporal gyrus to the lateral anterior parietal (postcentral) cortex. Smaller clusters were located in the left and right middle frontal gyrus, right thalamus, lateral occipital area, middle/posterior cingulate extending to precuneus.

### Positive and negative correlation of narrative components with the dialogue

**Fig 4:**
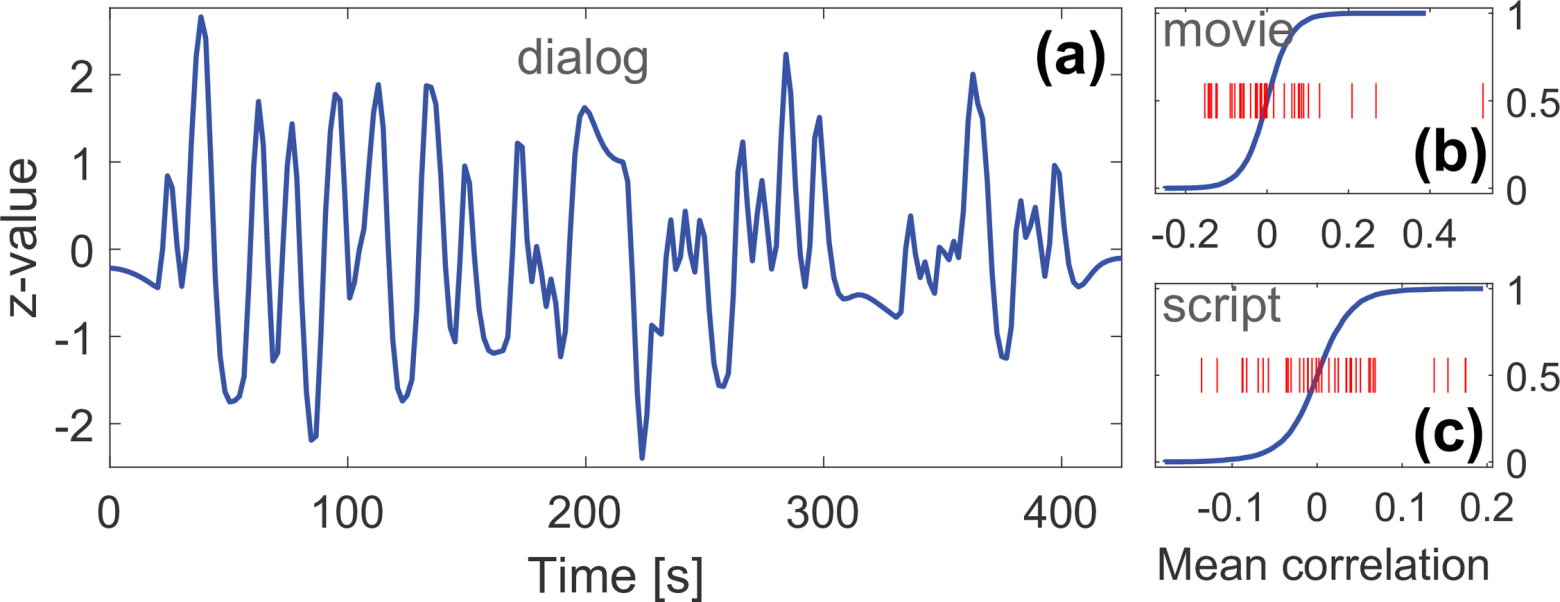
Time-course of the dialog regressor and correlation coefficients with ICs in movie and script conditions. (a): Time-course of the preprocessed dialog regressor that was compared against IC time-courses. (b)-(c): Mean correlations coefficients (red lines; one for each component, 40 values) between the dialog regressor and IC time-courses for (b) movie and (c) script condition plotted against the corresponding cumulative empirical null-distributions (blue lines).

The temporal correlation analysis between the dialog regressor and ICs revealed statistically significant mean correlations (p<0.05, FDR adjusted over 40 components) for total 7 different components. For the movie condition, these were IC4 (0.53; mean correlation) and two not TOP-5 components (0.21 and 0.27). For the script condition, these were IC4 (0.15), IC1 (-0.14) and three not TOP-5 components (0.17, 0.17, and 0.14). IC4, thus, correlated positively with the dialog in both modalities. On the other hand, IC1 correlated negatively in both conditions, although the correlation was not significant (-0.14; p ≈ 0.02 uncorrected) for the movie condition. These results indicate that of TOP-5 components, IC4 was *activated* and IC1 was *deactivated* in the presence of the dialog, either spoken or written. The remaining five, not TOP-5 components, mainly covered default mode and precuneus networks and auditory cortices. Due to their lower between-condition correlations (i.e., p>0.05, FDR adjusted) and ranking positions, they were not considered narrative driven. The preprocessed dialog regressor (i.e., convolved, filtered and z-scored; see Methods), mean correlation coefficients and empirical null-dictributions are depicted in Fig 4.

## Discussion

While cinema’s attraction largely derives from narratives that depict a range of familiar, yet significant socio-emotional situations that the viewers can recognize and share, same phenomenon takes place when reading novels, or listening to audio drama. Making sense of narrative contents involves semantic associations, memory and self-reflection, contextualization, management of longer temporal sequences, and so on. In line with hierarchical models of narrative comprehension [3,5] and high synchrony between narrative events and neural activity in fronto-parietal networks [12], we expected the modality-invariance to increase from the lower-order sensory regions towards high order cognitive regions in the parietal, temporal and frontal areas.

Our TOP-5 covered not only various cortical and subcortical high order cognitive areas, but also limbic areas. According to NeuroSynth meta-analysis decoder [21], our IC1 was associated particularly with default mode and memory functions (episodic and autobiographical) and IC4 was associated with speech, sentence, and language related functions. This was further reinforced by the fact that IC4 component was significantly correlated with the dialog predictor. Our TOP-5 narrative components partially overlapped with the theory-of-mind network [22–24], default mode network [25] and regions associated with social interaction [26,27]. In particular, IC1, IC2 and IC5, included parietal inferior and right angular gyrus which have been associated with theory of mind processing [23,28,29]. These ICs also contained regions, such as precuneous, parahippocampal gyrus (PHG), medial prefrontal and cingulate cortices, which are central for default mode network [25]. It has been suggested that default mode and theory of mind networks are related because of their role in social cognition [30]. In this sense, spatial overlap is not surprising in context of our strongly emotional stimulus centered on social interaction; the viewer needs to infer intents and beliefs of characters and reflect those to their behavior and emotions, as well as anticipate their future actions. Familiar story elements, including a family dinner and social conflicts, presumably have autobiographical memory associations for many subjects. These cognitive functions have been linked to both to theory of mind and default mode networks [31,32]. We also found right lateralization in our TOP-5 network, particularly for IC5 and IC2 components. This is in line with the previous studies that have assigned the discourse processing [33], or broader, more coarse (unsecure) inference [34] to the right hemisphere.

## Narrative networks and comparison with previous work

Unlike in Regev et al. [6], where voxel-wise ISC method was applied, we used spatial ICA. ICA takes advantage of the multivariate aspect of fMRI data by pinpointing such groups of voxels whose activation patterns are statistically independent from each other, thus avoiding strict voxel-wise temporal similarity requirement of ISC. We deem that such flexibility regarding the spatial locations is especially relevant for complex neural processes, such as narrative comprehension. This notion agrees with the previous studies (see, e.g., [35]), which have demonstrated the highest inter-subject variability of functional connectivity in the parietal, frontal and temporal association cortices that are considered essential to complex cognitive functions [36]. ICA (with GIFT toolbox implementation) has proven to give reliable and robust results when compared to ISC and the more traditional general linear model approach [1,11,37–39]. Despite methodological differences, our study supports the main findings in [3,5,6] that narrative comprehension occurs in distributed higher-order network covering frontal, temporal and superior parietal regions. Lower-order sensory components did not reach high cross-modality correlations in our analysis. This was expected because of large differences in visual and auditory properties of the two stimuli.

Furthermore, in contrast to the study of Regev et al. [6], where the modality comparison was done between two non-overlapping subject groups where each group was exposed to a different set of stimuli, in our experimental design all subjects experienced both stimuli conditions in the manner counterbalanced at the group level. Modality-specific signals were obtained by averaging over subjects before computing correlation between modalities, thus minimizing possible subject-specific biases (intrinsic signals) in the correlation values. This allowed us to better pinpoint stimulus-driven shared parts of the activation signal.

Our narrative TOP-5 components, which had the highest correlation between the two narrative stimulus conditions, showed a significant lack of primary visual areas presumable due to large visual differences in two conditions: The movie-viewing condition involved faces, human bodies, living spaces, dynamics, colors, and movements, while the script-reading involved static text slides with light-gray background and black text. Indeed, cross-modal correlations in primary visual and auditory cortices were found notably smaller for both ICA and atlas-based time-courses. Interestingly, the superior temporal gyrus (IC4) had high correlation between both conditions even though only the movie-viewing involved sound. According to NeuroSynth meta-analysis decoder [21], our IC4 was associated with speech, sentence, and language related functions. As these areas are known to engage during viewing social situations (posterior superior temporal sulcus, e.g., in [26]), the presence of the social interaction in our narrative could explain the overlap. It has also been suggested that “the multimodal mental experience of reading is in fact a heterogeneous complex of asynchronous neural responses, and that auditory and visual modalities often process distinct temporal frames of our environment at the same time” [40]. Such mechanism provides an alternative explanation: The script-reading in our experiment involved imagining audio events, especially speech, that could engage temporal areas typically associated with auditory perceptions similar to those viewed in the film. This alternative mechanism receives support from the fact that IC4 component was significantly correlated with the dialog predictor and coincides with the STS activation demonstrated for auditory narrative with longer temporal receptive windows (cf. Fig 2C–2E in [5]).

A key difference to language-based narrative presentations in Regev et al. [6] was that only one of our narrative presentations relied exclusively on verbal communication. Our IC4 component’s map remarkably resembles the map of brain activations common for the perception of speech and imagery of hearing of the visually presented word (see Figs 1 and 2 in [41]). In line with this finding, IC4 component demonstrated positive correlation with the dialogue predictor. In contrast, IC1 component demonstrated negative correlation with the dialogue regressor which points towards a different kind of language dependency. In the movie condition of our experiment the dialog was represented with natural speech. A previous fMRI study by Moreno et al. [42] employing natural and scrambled speech stimulus have revealed BOLD signal decrease in the precuneus, PHG, middle and inferior frontal gyri and parietal inferior lobule during natural speech comprehension. IC1 encompasses those regions and might therefore reflect its involvement in the comprehension of the natural speech (for movie dialogues) and imagery of the natural speech (for written dialogues). This further reassured us that our design enabled pinpointing a narrative-comprehension network IC1, which would not have been possible with a stimulus containing spoken or written language alone.

Our main motivation for the dialog regression analysis was to rule out the most obvious reason (dialog) behind cross-modality correlations. However, there are likely other narrative-related regressors that would produce high correlations with other TOP-5 components, not just IC4. Two of this type of regressors, plot suspension and cognitive demand, were used in a study by Naci et al. [12] where significant correlations were found with ICs in fronto-parietal regions. Such high-level regressors cannot be directly extracted from the low-level/physical features of the stimulus, but require human annotators to take into account complex nuances in narratives (e.g., non-linear effects, including history dependency). Employment of such carefully chosen “high-level” regressors might have provided additional insight into the factors behind TOP-5 cross-modal synchronization, but was beyond the primary focus and scope of the current study.

Taken together, our TOP-5 components had a high correspondence with narrative-responsive regions reported in previous fMRI studies involving narratives [5–7,12]. In addition, our IC1 contains various new regions, such as PHG, anterior cingulate cortex, and superior and middle frontal gyri, that were not previously reported. Cingulate cortex and PHG in particular have a central role in contextual memory, which is required in making semantic associations and contextualization to interpret character actions [43]. Making associations is central in interpreting our narrative, where audio-visual information (e.g., facial expressions and voice tones) and dialog are skillfully combined to hint the viewer for an upcoming climax. Inclusion of anterior cingulate cortex could also result from strong negative emotions in the story, which are known to activate this region [44], especially since our stimulus was not balanced in respect to negative and positive valence in particular. Besides additional regions, visual comparison of the maps (cf. Fig 3C in [6]), not just that of the region names and cluster peaks, reveals, however, strong resemblance with our IC1 network.

## Conclusions

By looking at brain networks’ temporal correlation across two modalities with the same narrative content, we identified five modality-invariant brain networks, which overlapped with theory-of-mind network [22–24] and language-based narrative comprehension networks [6]. The top-ranked modality-invariant network correlated negatively with the dialogue predictor confirming that we pinpointed a narrative-comprehension network that is not language-reliant. These findings provide new insight into narrative comprehension networks across different stimulus presentation modalities and substantially extend earlier results based on language-related paradigms.
